# Localization and movement of Tregs in gastrointestinal tract: a systematic review

**DOI:** 10.1186/s41232-022-00232-8

**Published:** 2022-11-03

**Authors:** Yosuke Harada, Kentaro Miyamoto, Akihiko Chida, Anna Tojo Okuzawa, Yusuke Yoshimatsu, Yumi Kudo, Tomohisa Sujino

**Affiliations:** 1 Department of Gastroenterology and Hepatology, School of Medicine, Keio University, Tokyo, Japan; 2 Miyarisan Pharm. Co. Ltd, Tokyo, Japan; 3 Department of Pediatric Surgery, School of Medicine, Keio University, Tokyo, Japan; 4 Center for the Diagnostic and Therapeutic Endoscopy, School of Medicine, Keio University, Tokyo, Japan

**Keywords:** Regulatory T cells (Tregs), Live imaging, Digestive tract, Multiphoton microscopy, Review

## Abstract

**Background:**

The intestine is rich in food-derived and microbe-derived antigens. Regulatory T cells (Tregs) are an essential T-cell population that prevents systemic autoimmune diseases and inhibits inflammation by encountering antigens. Previously, it was reported that the functional loss of Tregs induces systemic inflammation, including inflammatory bowel disease and graft-versus-host disease in human and murine models. However, there is a dearth of information about how Tregs localize in different tissues and suppress effector cells.

**Main body:**

The development of Tregs and their molecular mechanism in the digestive tract have been elucidated earlier using murine genetic models, infectious models, and human samples. Tregs suppress immune and other nonimmune cells through direct effect and cytokine production. The recent development of *in vivo* imaging technology allows us to visualize how Tregs localize and move in the settings of inflammation and homeostasis. This is important because, according to a recent report, Treg characterization and function are regulated by their location. Tregs located in the proximal intestine and its draining lymph nodes induce tolerance against food antigens, and those located in the distal intestine suppress the inflammation induced by microbial antigens. Taken together, various Tregs are induced in a location-specific manner in the gastrointestinal tract and influence the homeostasis of the gut.

**Conclusion:**

In this review, we summarize how Tregs are induced in the digestive tract and the application of *in vivo* Treg imaging to elucidate immune homeostasis in the digestive tract.

**Supplementary Information:**

The online version contains supplementary material available at 10.1186/s41232-022-00232-8.

## Background

Regulatory T cells (Tregs) are important for maintaining immune tolerance for self-antigens and suppressing inflammation. Loss of Tregs function induces autoimmune diseases and disrupts the overall homeostasis [1–4]. Tregs in inflammatory bowel disease (IBD) patients were less able to suppress the effector cells in the lamina propria [5, 6]. Moreover, the risk of graft-versus-host disease (GVHD) following bone marrow transplantation was associated with the depletion of Tregs in peripheral blood, while the introduction of Tregs into GVHD mice improved their survival [7, 8].

The importance of Tregs was elucidated using mice and human forkhead box p3 (*Foxp3*) gene mutation studies; human IPEX (immune dysregulation, polyendocrinopathy, enteropathy, X-linked) syndrome, caused by *Foxp3* mutation, induces early onset of T-cell-dependent lymphoproliferation with cytokine storm [9–12].

The mechanism of Treg suppression of other immune cells was studied extensively [13, 14]. Tregs express a substantial number of genes, including those of secreted proteins and molecules, on the cell surface. They suppress other immune cells via cell contact independent and dependent mechanisms. The suppression via a cell contact independent mechanism is through the secretion of the major cytokines, such as IL-10, IL-35, granzyme B, and TGFβ [15–17]. Lack of IL-10 and IL-35 production by Tregs induces inflammation in the colon and lungs [18, 19]. TGFβ, which is essential for the induction of the Tregs, suppresses helper T-cell 1 (Th1) response [20, 21].

**Fig. 1:**
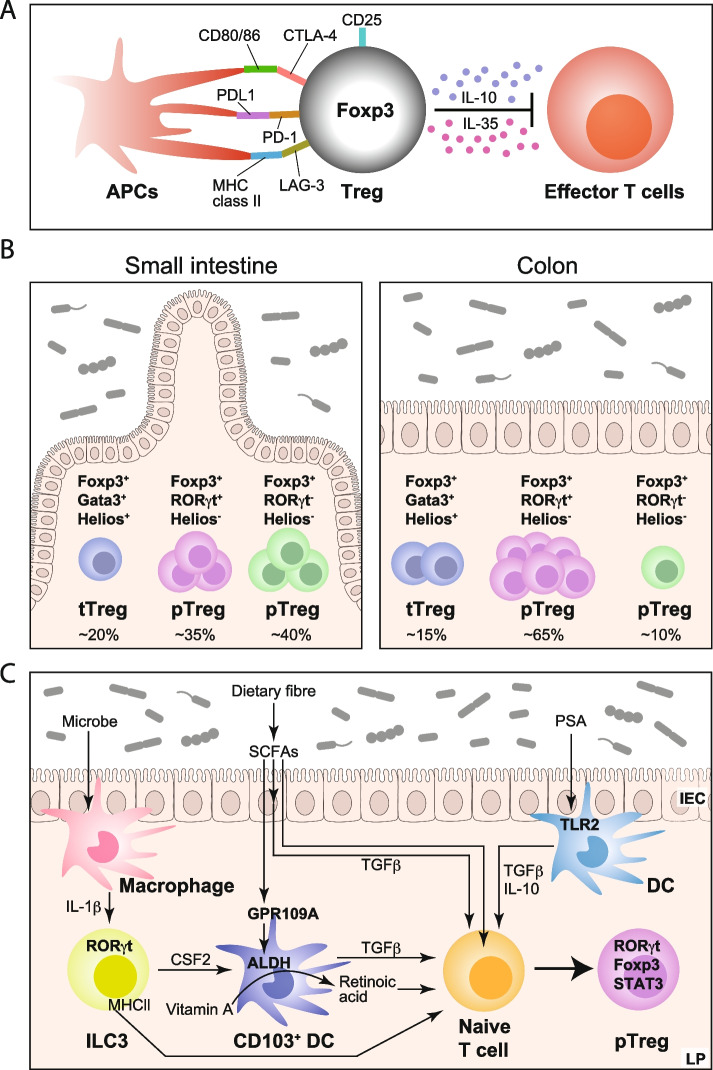
Characteristics of Treg in gut. **A** Tregs suppress effector cells and antigen-presenting cells via surface protein and cytokine. **B** Helios^+^Gata3^+^Foxp3^+^Tregs (thymic Tregs: tTregs), Helios^−^Rorγt^+^Foxp3^+^Tregs (peripheral Tregs: pTregs), Helios^−^Rorγt^−^Foxp3^+^Tregs (peripheral Tregs: pTregs) in small intestine (left) and colon (right). The percentage of each population in total Tregs was listed below. **C** Schema of generation of peripheral Tregs in gut

Tregs-induced suppression via cell contact-dependent mechanism is performed through the major molecules expressed on Tregs surface, such as IL-2 receptor (IL-2R), CTLA-4, PD-1, LAG-3, GITR, and TIGIT. CD25, known as the original Treg cell marker, is highly expressed by Tregs (Fig. 1A). High levels of IL-2R expression deprive effector T cells of IL-2, resulting in inhibition of proliferation [22]. CTLA-4 downregulates the CD80/CD86 expression on the antigen-presenting cells (APCs) [23, 24]. Accordingly, patients with CTLA-4 haploinsufficiency have impaired Treg functions, leading to the development of Crohn’s-like intestinal inflammation [25–27]. Moreover, patients treated with anti-CTLA-4 antibodies developed colitis as a side effect.

The PD-1 co-inhibitory receptor is highly expressed on Tregs. PD-1-mediated signaling inhibits CD28 costimulation by binding PD ligands 1 and 2, resulting in the inhibition of T-cell costimulation in the early phase after antigen stimulation [28].

LAG-3, which binds MHC class 2, is required for the suppressive activity of Tregs [29]. LAG-3 suppresses IL-23 on Cx3cr1^+^ macrophage, enhancing IL-22 production from group 3 innate lymphoid cells in an anti-CD40 colitis model [30]. TIGIT promotes IL-10 production on APCs [31]. GITR, one of the TNF receptor family members, is highly expressed on Tregs [32, 33].

More details about the functioning of Tregs have been elucidated recently, yet their action *in vivo* is still unknown. We review how the Tregs are generated and summarize Treg localization and movement in the gut using an *in vivo* imaging system.

## Importance of Tregs in the gut

Tregs develop in either the thymus or peripheral tissue. Tregs that develop in the thymus are called “tTregs,” and transcription factors such as Helios or Neuropilin are used to identify tTregs [34–40]. The other Tregs originating extrathymically in peripheral tissues are called “pTregs.” pTregs develop from T conventional cells (Foxp3^−^ cells) [41, 42]. Based on their location and function, pTregs can be classified into three groups: central, effector, and tissue-resident Tregs. Central Tregs that express CD62L^high^ and CCR7^+^ are the major population of naïve Tregs, and they localize in secondary lymph nodes. Effector Tregs are CD62L^low^ CCR7^low^ Tregs. Tissue-resident Tregs reside in nonlymphoid organs, especially the colon. Most of the Tregs in the gut in a steady state are tissue-resident pTregs [13, 43]. Like conventional T cells, Tregs are governed by specific transcriptional factors. T-bet^+^ Tregs, IRF4^+^ Tregs, and STAT3^+^ Tregs suppress Tbet^+^ T cells (Th1), IRF4^+^ T cells (Th2), and STAT3^+^ T cells (Th17), respectively [44–46]. Other Tregs express unique transcription factors to adapt to their microenvironment. For instance, some GATA3^+^Helios^+^Tregs expanded during tissue damage response to IL-33 [47, 48].

In the gut, some of the pTregs express the transcriptional factor retinoic acid-related orphan receptor-γt (Rorγt), which was initially described as the essential transcriptional factor for Th17 cell development [49] (Fig. 1B).

Rorγt^+^ pTregs develop under the existence of microbial antigens; they inhibit Th17 response during gut inflammation [50]. Rorγt^−^ Helios^−^ pTregs are induced by dietary antigens [51]. According to a recent report, Treg characterization and function are regulated by location. Tregs located in the proximal intestine and its draining lymph node induce tolerance against the food antigens, while those in the distal intestine suppress inflammation induced by the microbial antigens [52] (mentioned in the “Treg localization and movement in the gut,” see Fig. 1C). Taken together, various Tregs are induced in a location-specific manner in the gastrointestinal tract.

## Treg *in vivo* imaging

**Fig. 2:**
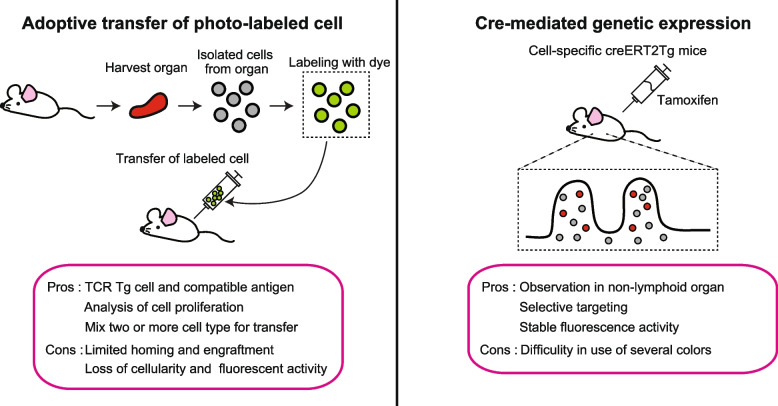
Two different techniques for *in vivo* imaging. In the procedure shown to the left, cells are isolated from organs harvested from mice, labeled with a specific dye, and transferred into mice. On the other hand, the procedure shown on the right establishes mice transgenic with the creERT2 gene and fluorescent protein targeting specific cells. The mice are then injected with the hormonal substance tamoxifen, which induces the activity-dependent expression of the fluorescent protein by Cre recombinase, allowing *in vivo* observation. Pros and cons for each method are listed at the lower part of the figure

CD4 imaging of lymphoid tissue is used to be challenging; now, two-photon intravital microscopy makes *in vivo* imaging possible (Fig. 2). Two-photon imaging enables us to visualize how the antigen is transferred from DCs to T cells in lymph nodes [53]. To visualize Tregs *in vivo*, two-photon microscopy was used to visualize CFSE-labeled CD4^+^CD25^+^T cells in lymphoid tissues [54, 55]. An antigen-specific model was developed to demonstrate that the contact of Tregs with dendritic cells (DCs) is required to inhibit Th cell activation. Imaging analysis revealed that Tregs in lymph nodes stopped for longer durations when they encountered antigen-bearing DCs and did not interact with CD4 non-Tregs. These data suggest that Tregs directly suppress the function of APCs, resulting in the inhibition of Th cell activation. CFSE-labeled CD4^+^CD25^+^T cells are injected during *in vivo* imaging to visualize the connection between other immune cells and cell proliferation. This system enables us to visualize Treg movement in an antigen-specific model. However, it is difficult to envisage where transferred cells do not migrate to, as the florescent activity fades in a time-dependent manner. Moreover, ex vivo cultured CFSE-labeled CD4^+^CD25^+^T cells may not have the same movement as internal Tregs.

To visualize live internal Tregs in tissue, we developed the tamoxifen-inducible *Foxp3*^eGFPcreERT2^: *Rosa26*^tdTomato^ mice [56, 57]. Tamoxifen can visualize the bona fide Tregs. Some unstable Tregs differentiate to other cells such as Th17, Th1, and CD8a expressing cells in the peripheral tissue, called exTregs [2, 57–61]. We visualized Tregs after 24 h of the tamoxifen induction, as more than 99% of tomato-positive cells still expressed Foxp3 protein [56, 57]. The benefit of this model is to visualize the internal bona fide Tregs, thus enabling us to image nonlymphoid organs. However, it is not quite feasible to image Tregs and immune/nonimmune cells simultaneously.

### Treg localization and movement in the gut

The distribution of Tregs in the gastrointestinal tract was different among different organs [62–65].

#### Stomach

**Fig. 3:**
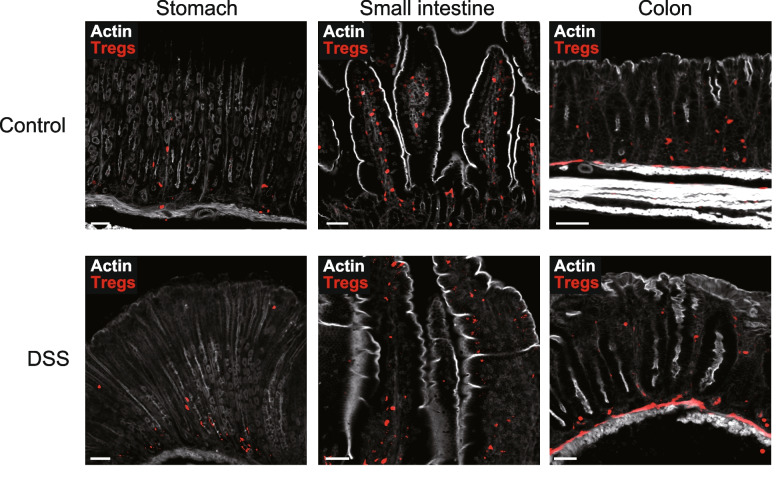
The distribution of Tregs in the gastrointestinal tract. (upper panel) Localization of tdTomato^+^ Tregs (red) within the stomach (left), small intestine (middle), and colon (right). (lower panel) Localization of tdTomato^+^ Tregs (red) within stomach (left), small intestine (middle), and colon (right) after 7-day DSS administration. Vibratome sections of each tissues from *Foxp3*^eGFPcreERT2^*Rosa26*^tdTomato^ mice were stained with phalloidin to show actin (white)

The role of Tregs in the stomach is not well understood. Infection with *Helicobacter* spp. is the trigger for chronic gastric inflammation, resulting in gastric cancer. However, in the mouse model, *Helicobacter* spp. induced inflammation in the small intestine and colon, especially in IL-10 knockout mice [66–68]. Tregs were observed to localize near the bottom of the glandular epithelium but not near the luminal side (Fig. 3). Further research is required to understand the function of Tregs in the stomach.

#### Small intestine

Oral antigen ovalbumin (OVA) is the commonly used model food antigen to understand Treg induction. Oral tolerance to OVA is dependent on the resident intestinal DCs, resulting in the induction of Tregs in the small intestine [69]. CD103^+^ DCs have more potential to induce Tregs than CD103^−^ DCs do, as CD103^+^ DCs highly express retinal aldehyde dehydrogenase (RALDH) [70]. The enriched retinol, vitamin A, and its metabolite (retinoic acid (RA)) are highly concentrated in the small intestine. RA produced by the enzyme RALDH is essential for the induction of Tregs [21]. Moreover, TGFβ itself induces CD11b^+^CD103^+^ DCs.

The distribution of the DC subsets is different in the small intestine and colon. The proportion of CD11b^+^CD103^+^ DCs is higher in the small intestine than that in the colon, whereas the CD11b^−^CD103^+^ DCs are the major population in the colon [63]. Consistent with the abundance of CD11b^+^CD103^+^ DCs in the small intestine, RALDH activity is the highest in the small intestine. These findings highlight the fact that the induction of Tregs in the small intestine is established by the relationship between the CD103^+^DCs and environmental factors, including RA and TGFβ.

TGFβ is produced by cells other than CD103^+^DCs. For instance, TLR2^+^ DCs produce TGFβ and IL-10 by polysaccharide A (PSA), and epithelial cells (ECs) also produce TGFβ by short-chain fatty acids (SCFAs) (i.e., metabolite of dietary fiber) [71, 72]. SCFAs also act directly on CD103^+^DCs via GPR109A [13, 73]. Recently, Rorγt^+^ innate lymphoid cells (ILC3) directly and indirectly produce Rorγt^+^ Tregs. ILC3 are activated by IL-1β^+^ macrophage [30]. MHC class 2-positive ILC3 directly induce Rorγt^+^ Tregs in colon, while CSF2 production from ILC3 indirectly induces Rorγt^+^ Tregs through the activation of CD103^+^DCs [74–77].

A small number of Tregs in the small intestine are localized in the intraepithelial compartment, while most Tregs in the small intestine are localized in the lamina propria. Moreover, unlike stomach and colonic Tregs, Tregs in the small intestine are localize through the middle of the villi to the bottom (Fig. 3).

#### Large intestine

Colonic Tregs are mainly induced by microbes and their metabolites. Germ-free mice have fewer colonic Tregs compared with specific pathogen-free mice [41, 42]. *Clostridia* species induce colonic Tregs [78]. SFCAs, such as butyrate, are a fermentation by-product of fiber digestion by commensal bacteria [79].

In a recent study, T cells in the thymus recognize colonic microbe antigen, indicating that parts of the colonic Tregs are generated in the thymus [80, 81]. Colonic Treg TCRs cloned into hybridomas reactive against fecal extract were found in thymic Tregs; this supports the conjecture that some colonic Tregs are of thymic origin [82]. Colonic Tregs are located around the bottom of the crypts (Fig. 3C).

*In vivo* imaging enables us to track the cell movement in each tissue (movie 1). Tregs in the small intestine move in the lamina propria at a velocity of 40 μm/s, and Tregs in the colon move at a velocity of 20–30 μm/s. It is unclear why Tregs migrate at various speeds in different tissues, but one possibility may be the difference in the spaces through which they migrate among different organs; another possibility may be due to the space in which they contact with surrounding immune and nonimmune cells.

TCRγδ T cells, abundant in the intraepithelial compartment, move at a speed of 30 μm/s. TCRγδ T cells interact with the intestinal epithelial cells (IECs) to detect microbe invasion and IEC damage, such as colon cancer [83, 84]. However, it remains unclear how Tregs interact with APCs and how they suppress other immune cells.

### Treg localization and intestinal disease

The 3D construction of Tregs in the peripheral tissue reveals that Tregs suppress not only immune cells but also other types of cells as well, such as fibroblasts, ECs, and neurons [85, 86]. Some Tregs are located near the intestinal stem cells, which induce IL-10 to sustain the intestinal stem cells. Some Tregs located near the enteric nerve and neuronal IL-6 induce the Rorγt^+^ Tregs to sustain the intestinal homeostasis [87].

IBD, including ulcerative colitis and Crohn’s disease, is characterized by chronic inflammation of the gastrointestinal tract. Although its exact mechanism is still unknown, the colitis mouse model suggests the importance of Tregs in colitis. Tregs are essential for the inhibition of colitis in the T-cell-adoptive transfer model [2, 88, 89]. IL-10-deficient mice spontaneously developed colitis [90, 91]. Despite the surge in the number of Tregs in the inflamed tissues [92], the suppressive function of Tregs is defected in these tissues.

The dextran sulfate sodium (DSS) model induced the localization of Tregs at the site of the chemical epithelial cell damage (Fig. 3). The localization of Tregs in the stomach, small intestine, and large intestine was not so different under DSS administration. Furthermore, previous studies observed that there was no change in the number of Tregs after DSS administration [2, 89]. These findings suggest that the acute epithelial damage itself does not immediately alter the localization of Tregs. We analyzed Treg localization in the early stages of the epithelial damage but not in the late or recovery stages. Moreover, we did not induce other types of inflammation, such as *Citrobacter *infection or adoptive T-cell transfer models. Accordingly, further research is required to decipher how their localization is manipulated.

As previously mentioned, intestinal Tregs are generated by environmental cues (see “Treg localization and intestinal disease”); other nonimmune cells also contribute to the induction of Tregs. Stromal cells produce microbiome-dependent RA [93]. IECs educate the immune system, especially tolerogenic DCs by TGFβ and RA, to induce Tregs in humans [94].

**Fig. 4:**
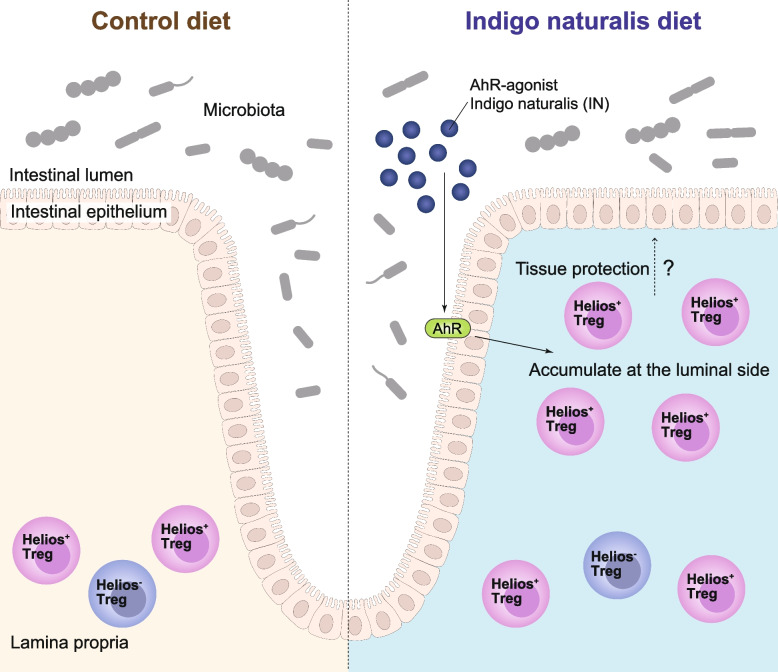
Treg location in steady-state and AHR-ligand diet condition

Aryl-hydrocarbon receptor (AhR) agonist, which is fermented by green vegetables, is one of the pivotal factors in maintaining gut homeostasis. Lack of the AhR signal induces chronic inflammation in the colon [95]. Indigo naturalis (IN) is an AhR agonist containing indole derivatives, such as indigo, indirubin, and indole-3-aldehyde. IN diet increased GATA3^lo^ Helios^+^ Rorγt^−^ Tregs in the colon (IN-Tregs). Intriguingly, these IN-Tregs were located next to MHC class 2-positive ECs near the luminal side in IN-fed mice, and they moved faster than the colonic Tregs in normal diet-fed mice [56] (Fig. 4). Thus, *in vivo* imaging will help decipher how the cells migrate not only in the steady state but also in the treatment or disease state.

## Conclusion

We summarized the localization and movement of Tregs in the gut, especially in the small intestine and colon. The novel technique of live imaging and the genetic development of animals have enabled us to visualize cell movement and localization. Integrating analysis of the localization/movement of immune cells with their function is required in the future. The next step in visualizing the condition of the cells will help us to understand how the immune homeostasis is regulated *in vivo*.

## Supplementary Information

**Additional file 1.** Movie 1. Intra-vital microscopy imaging of Tregs in small intestine and colon.

## Data Availability

All the data and materials are stocked in the Keio University.

## Abbreviations

Foxp3: Forkhead box p3
IBD: Inflammatory bowel disease
IL: Interleukin
IPEX: Immune dysregulation, polyendocrinopathy, enteropathy, and X-linked
TGFβ: Transforming growth factor β
Tregs: Regulatory T cells
Th: T-helper cells
IL-2R: IL-2 receptor
APCs: Antigen-presenting cells
MHC: Major histocompatibility complex
TNF: Tumor necrosis factor
Rorγt: Retinoid-related orphan receptor gamma t
DCs: Dendritic cells
OVA: Oral antigen ovalbumin
CTLA-4: Cytotoxic lymphocyte antigen 4
PD-1: Programmed death receptor 1
LAG3: Latent activation gene 3
RALDH: Retinal aldehyde dehydrogenase
RA: Retinoic acid
GF: Germ-free
SPF: Specific pathogen-free
SFCAs: Short-chain fatty acids
TCRs: T-cell receptor
AhR: Aryl-hydrocarbon receptor
IN: Indigo naturalis
ECS: Epithelial cells

## References

[1] Asano MS, Ahmed R (1996). CD8 T cell memory in B cell-deficient mice. J Exp Med.

[2] Sujino T, Kanai T, Ono Y, Mikami Y, Hayashi A, Doi T, Matsuoka K, Hisamatsu T, Takaishi H, Ogata H (2011). Regulatory T cells suppress development of colitis, blocking differentiation of T-helper 17 into alternative T-helper 1 cells. Gastroenterology.

[3] Morrissey PJ, Charrier K, Braddy S, Liggitt D, Watson JD (1993). CD4+ T cells that express high levels of CD45RB induce wasting disease when transferred into congenic severe combined immunodeficient mice. Disease development is prevented by cotransfer of purified CD4+ T cells. J Exp Med.

[4] Powrie F, Mason D (1990). OX-22high CD4+ T cells induce wasting disease with multiple organ pathology: prevention by the OX-22low subset. J Exp Med.

[5] Maul J, Loddenkemper C, Mundt P, Berg E, Giese T, Stallmach A, Zeitz M, Duchmann R (2005). Peripheral and intestinal regulatory CD4+ CD25(high) T cells in inflammatory bowel disease. Gastroenterology.

[6] Fantini MC, Rizzo A, Fina D, Caruso R, Sarra M, Stolfi C, Becker C, Macdonald TT, Pallone F, Neurath MF (2009). Smad7 controls resistance of colitogenic T cells to regulatory T cell-mediated suppression. Gastroenterology.

[7] Trzonkowski P, Zaucha JM, Mysliwska J, Balon J, Szmit E, Halaburda K, Bieniaszewska M, Mlotkowska M, Hellmann A, Mysliwski A (2004). Differences in kinetics of donor lymphoid cells in response to G-CSF administration may affect the incidence and severity of acute GvHD in respective HLA-identical sibling recipients. Med Oncol.

[8] Taylor PA, Lees CJ, Blazar BR (2002). The infusion of ex vivo activated and expanded CD4(+)CD25(+) immune regulatory cells inhibits graft-versus-host disease lethality. Blood.

[9] Chatila TA, Blaeser F, Ho N, Lederman HM, Voulgaropoulos C, Helms C, Bowcock AM (2000). JM2, encoding a fork head-related protein, is mutated in X-linked autoimmunity-allergic disregulation syndrome. J Clin Investig.

[10] Brunkow ME, Jeffery EW, Hjerrild KA, Paeper B, Clark LB, Yasayko SA, Wilkinson JE, Galas D, Ziegler SF, Ramsdell F (2001). Disruption of a new forkhead/winged-helix protein, scurfin, results in the fatal lymphoproliferative disorder of the scurfy mouse. Nat Genet.

[11] Bennett CL, Christie J, Ramsdell F, Brunkow ME, Ferguson PJ, Whitesell L, Kelly TE, Saulsbury FT, Chance PF, Ochs HD (2001). The immune dysregulation, polyendocrinopathy, enteropathy, X-linked syndrome (IPEX) is caused by mutations of FOXP3. Nat Genet.

[12] Wildin RS, Ramsdell F, Peake J, Faravelli F, Casanova JL, Buist N, Levy-Lahad E, Mazzella M, Goulet O, Perroni L (2001). X-linked neonatal diabetes mellitus, enteropathy and endocrinopathy syndrome is the human equivalent of mouse scurfy. Nat Genet.

[13] Jacobse J, Li J, Rings E, Samsom JN, Goettel JA (2021). Intestinal regulatory T cells as specialized tissue-restricted immune cells in intestinal immune homeostasis and disease. Front Immunol.

[14] Josefowicz SZ, Lu LF, Rudensky AY (2012). Regulatory T cells: mechanisms of differentiation and function. Annu Rev Immunol.

[15] von Boehmer H (2005). Mechanisms of suppression by suppressor T cells. Nat Immunol.

[16] Grossman WJ, Verbsky JW, Barchet W, Colonna M, Atkinson JP, Ley TJ (2004). Human T regulatory cells can use the perforin pathway to cause autologous target cell death. Immunity.

[17] Gondek DC, Lu LF, Quezada SA, Sakaguchi S, Noelle RJ (2005). Cutting edge: contact-mediated suppression by CD4+CD25+ regulatory cells involves a granzyme B-dependent, perforin-independent mechanism. J Immunol.

[18] Rubtsov YP, Rasmussen JP, Chi EY, Fontenot J, Castelli L, Ye X, Treuting P, Siewe L, Roers A, Henderson WR (2008). Regulatory T cell-derived interleukin-10 limits inflammation at environmental interfaces. Immunity.

[19] Collison LW, Workman CJ, Kuo TT, Boyd K, Wang Y, Vignali KM, Cross R, Sehy D, Blumberg RS, Vignali DA (2007). The inhibitory cytokine IL-35 contributes to regulatory T-cell function. Nature.

[20] Li MO, Wan YY, Flavell RA (2007). T cell-produced transforming growth factor-beta1 controls T cell tolerance and regulates Th1- and Th17-cell differentiation. Immunity.

[21] Mucida D, Park Y, Kim G, Turovskaya O, Scott I, Kronenberg M, Cheroutre H (2007). Reciprocal TH17 and regulatory T cell differentiation mediated by retinoic acid. Science (New York, NY).

[22] Pandiyan P, Zheng L, Ishihara S, Reed J, Lenardo MJ (2007). CD4+CD25+Foxp3+ regulatory T cells induce cytokine deprivation-mediated apoptosis of effector CD4+ T cells. Nat Immunol.

[23] Wing K, Onishi Y, Prieto-Martin P, Yamaguchi T, Miyara M, Fehervari Z, Nomura T, Sakaguchi S (2008). CTLA-4 control over Foxp3+ regulatory T cell function. Science.

[24] Qureshi OS, Zheng Y, Nakamura K, Attridge K, Manzotti C, Schmidt EM, Baker J, Jeffery LE, Kaur S, Briggs Z (2011). Trans-endocytosis of CD80 and CD86: a molecular basis for the cell-extrinsic function of CTLA-4. Science.

[25] Schubert D, Bode C, Kenefeck R, Hou TZ, Wing JB, Kennedy A, Bulashevska A, Petersen BS, Schäffer AA, Grüning BA (2014). Autosomal dominant immune dysregulation syndrome in humans with CTLA4 mutations. Nat Med.

[26] Kuehn HS, Ouyang W, Lo B, Deenick EK, Niemela JE, Avery DT, Schickel JN, Tran DQ, Stoddard J, Zhang Y (2014). Immune dysregulation in human subjects with heterozygous germline mutations in CTLA4. Science.

[27] Zeissig S, Petersen BS, Tomczak M, Melum E, Huc-Claustre E, Dougan SK, Laerdahl JK, Stade B, Forster M, Schreiber S (2015). Early-onset Crohn's disease and autoimmunity associated with a variant in CTLA-4. Gut.

[28] Seidel JA, Otsuka A, Kabashima K (2018). Anti-PD-1 and anti-CTLA-4 therapies in cancer: mechanisms of action, efficacy, and limitations. Front Oncol.

[29] Huang CT, Workman CJ, Flies D, Pan X, Marson AL, Zhou G, Hipkiss EL, Ravi S, Kowalski J, Levitsky HI (2004). Role of LAG-3 in regulatory T cells. Immunity.

[30] Bauché D, Joyce-Shaikh B, Jain R, Grein J, Ku KS, Blumenschein WM, Ganal-Vonarburg SC, Wilson DC, McClanahan TK, Malefyt RW (2018). LAG3(+) regulatory T cells restrain interleukin-23-producing CX3CR1(+) gut-resident macrophages during group 3 innate lymphoid cell-driven colitis. Immunity.

[31] Yu X, Harden K, Gonzalez LC, Francesco M, Chiang E, Irving B, Tom I, Ivelja S, Refino CJ, Clark H (2009). The surface protein TIGIT suppresses T cell activation by promoting the generation of mature immunoregulatory dendritic cells. Nat Immunol.

[32] McHugh RS, Whitters MJ, Piccirillo CA, Young DA, Shevach EM, Collins M, Byrne MC (2002). CD4(+)CD25(+) immunoregulatory T cells: gene expression analysis reveals a functional role for the glucocorticoid-induced TNF receptor. Immunity.

[33] Shimizu J, Yamazaki S, Takahashi T, Ishida Y, Sakaguchi S (2002). Stimulation of CD25(+)CD4(+) regulatory T cells through GITR breaks immunological self-tolerance. Nat Immunol.

[34] Bilate AM, Lafaille JJ (2012). Induced CD4+Foxp3+ regulatory T cells in immune tolerance. Annu Rev Immunol.

[35] Thornton AM, Korty PE, Tran DQ, Wohlfert EA, Murray PE, Belkaid Y, Shevach EM (2010). Expression of Helios, an Ikaros transcription factor family member, differentiates thymic-derived from peripherally induced Foxp3+ T regulatory cells. J Immunol.

[36] Verhagen J, Wraith DC (2010). Comment on "expression of Helios, an Ikaros transcription factor family member, differentiates thymic-derived from peripherally induced Foxp3+ T regulatory cells". J Immunol.

[37] Sarris M, Andersen KG, Randow F, Mayr L, Betz AG (2008). Neuropilin-1 expression on regulatory T cells enhances their interactions with dendritic cells during antigen recognition. Immunity.

[38] Yadav M, Louvet C, Davini D, Gardner JM, Martinez-Llordella M, Bailey-Bucktrout S, Anthony BA, Sverdrup FM, Head R, Kuster DJ (2012). Neuropilin-1 distinguishes natural and inducible regulatory T cells among regulatory T cell subsets *in vivo*. J Exp Med.

[39] Weiss JM, Bilate AM, Gobert M, Ding Y, Curotto de Lafaille MA, Parkhurst CN, Xiong H, Dolpady J, Frey AB, Ruocco MG (2012). Neuropilin 1 is expressed on thymus-derived natural regulatory T cells, but not mucosa-generated induced Foxp3+ T reg cells. J Exp Med.

[40] Glinka Y, Prud'homme GJ (2008). Neuropilin-1 is a receptor for transforming growth factor beta-1, activates its latent form, and promotes regulatory T cell activity. J Leukoc Biol.

[41] Lathrop SK, Bloom SM, Rao SM, Nutsch K, Lio CW, Santacruz N, Peterson DA, Stappenbeck TS, Hsieh CS (2011). Peripheral education of the immune system by colonic commensal microbiota. Nature.

[42] Cebula A, Seweryn M, Rempala GA, Pabla SS, McIndoe RA, Denning TL, Bry L, Kraj P, Kisielow P, Ignatowicz L (2013). Thymus-derived regulatory T cells contribute to tolerance to commensal microbiota. Nature.

[43] Liston A, Gray DH (2014). Homeostatic control of regulatory T cell diversity. Nat Rev Immunol.

[44] Koch MA, Tucker-Heard G, Perdue NR, Killebrew JR, Urdahl KB, Campbell DJ (2009). The transcription factor T-bet controls regulatory T cell homeostasis and function during type 1 inflammation. Nat Immunol.

[45] Zheng Y, Chaudhry A, Kas A, deRoos P, Kim JM, Chu TT, Corcoran L, Treuting P, Klein U, Rudensky AY (2009). Regulatory T-cell suppressor program co-opts transcription factor IRF4 to control T(H)2 responses. Nature.

[46] Chaudhry A, Rudra D, Treuting P, Samstein RM, Liang Y, Kas A, Rudensky AY (2009). CD4+ regulatory T cells control TH17 responses in a Stat3-dependent manner. Science.

[47] Schiering C, Krausgruber T, Chomka A, Fröhlich A, Adelmann K, Wohlfert EA, Pott J, Griseri T, Bollrath J, Hegazy AN (2014). The alarmin IL-33 promotes regulatory T-cell function in the intestine. Nature.

[48] Wohlfert EA, Grainger JR, Bouladoux N, Konkel JE, Oldenhove G, Ribeiro CH, Hall JA, Yagi R, Naik S, Bhairavabhotla R (2011). GATA3 controls Foxp3^+^ regulatory T cell fate during inflammation in mice. J Clin Investig.

[49] Ivanov II, McKenzie BS, Zhou L, Tadokoro CE, Lepelley A, Lafaille JJ, Cua DJ, Littman DR (2006). The orphan nuclear receptor RORgammat directs the differentiation program of proinflammatory IL-17+ T helper cells. Cell.

[50] Sefik E, Geva-Zatorsky N, Oh S, Konnikova L, Zemmour D, McGuire AM, Burzyn D, Ortiz-Lopez A, Lobera M, Yang J (2015). Mucosal immunology individual intestinal symbionts induce a distinct population of RORγ^+^ regulatory T cells. Science.

[51] Kim KS, Hong SW, Han D, Yi J, Jung J, Yang BG, Lee JY, Lee M, Surh CD (2016). Dietary antigens limit mucosal immunity by inducing regulatory T cells in the small intestine. Science.

[52] Esterházy D, Canesso MCC, Mesin L, Muller PA, de Castro TBR, Lockhart A, ElJalby M, Faria AMC, Mucida D (2019). Compartmentalized gut lymph node drainage dictates adaptive immune responses. Nature.

[53] Pasqual G, Chudnovskiy A, Tas JMJ, Agudelo M, Schweitzer LD, Cui A, Hacohen N, Victora GD (2018). Monitoring T cell-dendritic cell interactions *in vivo* by intercellular enzymatic labelling. Nature.

[54] Tang Q, Adams JY, Tooley AJ, Bi M, Fife BT, Serra P, Santamaria P, Locksley RM, Krummel MF, Bluestone JA (2006). Visualizing regulatory T cell control of autoimmune responses in nonobese diabetic mice. Nat Immunol.

[55] Tadokoro CE, Shakhar G, Shen S, Ding Y, Lino AC, Maraver A, Lafaille JJ, Dustin ML (2006). Regulatory T cells inhibit stable contacts between CD4+ T cells and dendritic cells *in vivo*. J Exp Med.

[56] Yoshimatsu Y, Sujino T, Miyamoto K, Harada Y, Tanemoto S, Ono K, Umeda S, Yoshida K, Teratani T, Suzuki T (2022). Aryl hydrocarbon receptor signals in epithelial cells govern the recruitment and location of Helios(+) Tregs in the gut. Cell Rep.

[57] Sujino T, London M, Hoytema van Konijnenburg DP, Rendon T, Buch T, Silva HM, Lafaille JJ, Reis BS, Mucida D (2016). Tissue adaptation of regulatory and intraepithelial CD4^+^ T cells controls gut inflammation. Science..

[58] London M, Bilate AM, Castro TBR, Sujino T, Mucida D (2021). Stepwise chromatin and transcriptional acquisition of an intraepithelial lymphocyte program. Nat Immunol.

[59] Bilate AM, London M, Castro TBR, Mesin L, Bortolatto J, Kongthong S, Harnagel A, Victora GD, Mucida D (2020). T cell receptor is required for differentiation, but not maintenance, of intestinal CD4(+) intraepithelial lymphocytes. Immunity.

[60] Harada Y, Sujino T, Miyamoto K, Nomura E, Yoshimatsu Y, Tanemoto S, Umeda S, Ono K, Mikami Y, Nakamoto N (2022). Intracellular metabolic adaptation of intraepithelial CD4(+)CD8αα(+) T lymphocytes. iScience.

[61] Miyao T, Floess S, Setoguchi R, Luche H, Fehling HJ, Waldmann H, Huehn J, Hori S (2012). Plasticity of Foxp3(+) T cells reflects promiscuous Foxp3 expression in conventional T cells but not reprogramming of regulatory T cells. Immunity.

[62] Mowat AM, Agace WW (2014). Regional specialization within the intestinal immune system. Nat Rev Immunol.

[63] Houston SA, Cerovic V, Thomson C, Brewer J, Mowat AM, Milling S (2016). The lymph nodes draining the small intestine and colon are anatomically separate and immunologically distinct. Mucosal Immunol.

[64] Li X, Zheng Y (2015). Regulatory T cell identity: formation and maintenance. Trends Immunol.

[65] Miragaia RJ, Gomes T, Chomka A, Jardine L, Riedel A, Hegazy AN, Whibley N, Tucci A, Chen X, Lindeman I (2019). Single-cell transcriptomics of regulatory T cells reveals trajectories of tissue adaptation. Immunity.

[66] Cahill RJ, Foltz CJ, Fox JG, Dangler CA, Powrie F, Schauer DB (1997). Inflammatory bowel disease: an immunity-mediated condition triggered by bacterial infection with Helicobacter hepaticus. Infect Immun.

[67] Denning TL, Kim G, Kronenberg M (2005). Cutting edge: CD4+CD25+ regulatory T cells impaired for intestinal homing can prevent colitis. J Immunol.

[68] Eden K (1960). Adoptive transfer colitis. Methods Mol Biol.

[69] Worbs T, Bode U, Yan S, Hoffmann MW, Hintzen G, Bernhardt G, Förster R, Pabst O (2006). Oral tolerance originates in the intestinal immune system and relies on antigen carriage by dendritic cells. J Exp Med.

[70] Esterházy D, Loschko J, London M, Jove V, Oliveira TY, Mucida D (2016). Classical dendritic cells are required for dietary antigen-mediated induction of peripheral T(reg) cells and tolerance. Nat Immunol.

[71] Mazmanian SK, Round JL, Kasper DL (2008). A microbial symbiosis factor prevents intestinal inflammatory disease. Nature.

[72] Smith PM, Howitt MR, Panikov N, Michaud M, Gallini CA, Bohlooly YM, Glickman JN, Garrett WS (2013). The microbial metabolites, short-chain fatty acids, regulate colonic Treg cell homeostasis. Science.

[73] Tao R, de Zoeten EF, Ozkaynak E, Chen C, Wang L, Porrett PM, Li B, Turka LA, Olson EN, Greene MI (2007). Deacetylase inhibition promotes the generation and function of regulatory T cells. Nat Med.

[74] Mortha A, Chudnovskiy A, Hashimoto D, Bogunovic M, Spencer SP, Belkaid Y, Merad M (2014). Microbiota-dependent crosstalk between macrophages and ILC3 promotes intestinal homeostasis. Science (New York, NY).

[75] Lyu M, Suzuki H, Kang L, Gaspal F, Zhou W, Goc J, Zhou L, Zhou J, Zhang W, Shen Z, et al. ILC3s select microbiota-specific regulatory T cells to establish tolerance in the gut. Nature. 2022;610(7933):744–51. 10.1038/s41586-022-05141-x PMC9613541 36071169

[76] Akagbosu B, Tayyebi Z, Shibu G, Paucar Iza YA, Deep D, Parisotto YF, Fisher L, Pasolli HA, Thevin V, Elmentaite R, et al. Novel antigen presenting cell imparts Treg-dependent tolerance to gut microbiota. Nature. 2022;610(7933):752–60. 10.1038/s41586-022-05309-5 PMC9605865 36070798

[77] Kedmi R, Najar TA, Mesa KR, Grayson A, Kroehling L, Hao Y, Hao S, Pokrovskii M, Xu M, Talbot J et al. A RORγt(+) cell instructs gut microbiota-specific T(reg) cell differentiation. Nature. 2022;610(7933):737–43. 10.1038/s41586-022-05089-y PMC9908423 36071167

[78] Atarashi K, Tanoue T, Shima T, Imaoka A, Kuwahara T, Momose Y, Cheng G, Yamasaki S, Saito T, Ohba Y (2011). Induction of colonic regulatory T cells by indigenous Clostridium species. Science.

[79] Furusawa Y, Obata Y, Fukuda S, Endo TA, Nakato G, Takahashi D, Nakanishi Y, Uetake C, Kato K, Kato T (2013). Commensal microbe-derived butyrate induces the differentiation of colonic regulatory T cells. Nature.

[80] Zegarra-Ruiz DF, Kim DV, Norwood K, Kim M, Wu WH, Saldana-Morales FB, Hill AA, Majumdar S, Orozco S, Bell R (2021). Thymic development of gut-microbiota-specific T cells. Nature.

[81] Michelson DA, Hase K, Kaisho T, Benoist C, Mathis D (2022). Thymic epithelial cells co-opt lineage-defining transcription factors to eliminate autoreactive T cells. Cell.

[82] Izraelson M, Nakonechnaya TO, Moltedo B, Egorov ES, Kasatskaya SA, Putintseva EV, Mamedov IZ, Staroverov DB, Shemiakina II, Zakharova MY (2018). Comparative analysis of murine T-cell receptor repertoires. Immunology.

[83] Reis BS, Darcy PW, Khan IZ, Moon CS, Kornberg AE, Schneider VS, Alvarez Y, Eleso O, Zhu C, Schernthanner M (2022). TCR-Vγδ usage distinguishes protumor from antitumor intestinal γδ T cell subsets. Science.

[84] Hoytema van Konijnenburg DP, Reis BS, Pedicord VA, Farache J, Victora GD, Mucida D (2017). Intestinal epithelial and intraepithelial T cell crosstalk mediates a dynamic response to infection. Cell.

[85] Huang H, Wang Z, Zhang Y, Pradhan RN, Ganguly D, Chandra R, Murimwa G, Wright S, Gu X, Maddipati R (2022). Mesothelial cell-derived antigen-presenting cancer-associated fibroblasts induce expansion of regulatory T cells in pancreatic cancer. Cancer Cell.

[86] Biton M, Haber AL, Rogel N, Burgin G, Beyaz S, Schnell A, Ashenberg O, Su CW, Smillie C, Shekhar K (2018). T helper cell cytokines modulate intestinal stem cell renewal and differentiation. Cell.

[87] Yan Y, Ramanan D, Rozenberg M, McGovern K, Rastelli D, Vijaykumar B, Yaghi O, Voisin T, Mosaheb M, Chiu I (2021). Interleukin-6 produced by enteric neurons regulates the number and phenotype of microbe-responsive regulatory T cells in the gut. Immunity.

[88] Powrie F, Leach MW, Mauze S, Caddle LB, Coffman RL (1993). Phenotypically distinct subsets of CD4+ T cells induce or protect from chronic intestinal inflammation in C. B-17 scid mice. Int Immunol.

[89] Ono Y, Kanai T, Sujino T, Nemoto Y, Kanai Y, Mikami Y, Hayashi A, Matsumoto A, Takaishi H, Ogata H (2012). T-helper 17 and interleukin-17-producing lymphoid tissue inducer-like cells make different contributions to colitis in mice. Gastroenterology.

[90] Fiorentino DF, Zlotnik A, Vieira P, Mosmann TR, Howard M, Moore KW, O'Garra A (1991). IL-10 acts on the antigen-presenting cell to inhibit cytokine production by Th1 cells. J Immunol.

[91] Huber S, Gagliani N, Esplugues E, O'Connor W, Huber FJ, Chaudhry A, Kamanaka M, Kobayashi Y, Booth CJ, Rudensky AY (2011). Th17 cells express interleukin-10 receptor and are controlled by Foxp3^-^ and Foxp3+ regulatory CD4+ T cells in an interleukin-10-dependent manner. Immunity.

[92] Tang Q, Bluestone JA, Kang SM (2012). CD4(+)Foxp3(+) regulatory T cell therapy in transplantation. J Mol Cell Biol.

[93] Vicente-Suarez I, Larange A, Reardon C, Matho M, Feau S, Chodaczek G, Park Y, Obata Y, Gold R, Wang-Zhu Y (2015). Unique lamina propria stromal cells imprint the functional phenotype of mucosal dendritic cells. Mucosal Immunol.

[94] Iliev ID, Spadoni I, Mileti E, Matteoli G, Sonzogni A, Sampietro GM, Foschi D, Caprioli F, Viale G, Rescigno M (2009). Human intestinal epithelial cells promote the differentiation of tolerogenic dendritic cells. Gut.

[95] Metidji A, Omenetti S, Crotta S, Li Y, Nye E, Ross E, Li V, Maradana MR, Schiering C, Stockinger B (2018). The environmental sensor AHR protects from inflammatory damage by maintaining intestinal stem cell homeostasis and barrier integrity. Immunity.

